# Barriers to the Pharmacologic Rescue of W1282X CFTR

**DOI:** 10.1021/acs.biochem.5c00590

**Published:** 2025-12-12

**Authors:** Candela Manfredi, Andras Rab, Disha Joshi, Ashlyn G. Winters, JaNise J. Jackson, Sam Molina, Michael Koval, Netaly Khazanov, Madison Jacobson, Kathryn Oliver, Hanoch Senderowitz, Eric J. Sorscher, Jeong S. Hong

**Affiliations:** † Department of Pediatrics, 12239 Emory University School of Medicine, Atlanta, Georgia 30322, United States; ‡ Experimental Models Core, Division of Pulmonary, Allergy, Critical Care and Sleep Medicine, Emory University School of Medicine, Atlanta, Georgia 30322, United States; § Department of Chemistry, 26731 Bar-Ilan University, Ramat-Gan 5290002, Israel

## Abstract

W1282X CFTR is the most prevalent CF-causing variant among cystic fibrosis patients of Ashkenazi descent and a mutational defect for which targeted drug therapy is not available. We show that administration of the potentiator VX-770 can augment levels of truncated W1282X CFTR in the plasma membrane, demonstrating that an established gating activator (i.e., “potentiator”) also rescues W1282X protein expression and surface localization (i.e., “corrector” function). Additionally, acute in vitro treatments with approved modulators VX-809 or VX-661 result in immediate potentiation of W1282X-dependent ion transport, showing that F508del CFTR correctors also augment W1282X CFTR channel activity. To investigate the mechanism, we tested a CFTR variant (G551D) exhibiting higher levels of CFTR-dependent potentiation following corrector treatment. Clinically approved CFTR correctors VX-445, VX-121, and VX-809 elicited potentiation of G551D CFTR. Forskolin dose dependence and molecular dynamic simulations indicated that corrector molecules promote acute CFTR gating by modifying protein conformation and enhancing heterodimerization of nucleotide binding domains, leading to potentiator-like effects. Although W1282X is poorly responsive to “readthrough” agents such as G418, the drug unexpectedly increases W1282X mRNA, augments surface-localized (truncated) protein, and promotes CFTR function, even in the absence of detectable stop codon suppression. Moreover, unlike other CFTR mutations such as F508del, proteasome blockade using ALLN partially rescues W1282X at the plasma membrane. These results highlight ways in which detailed mechanistic analysis and modulator profiling are needed to characterize CFTR mutations such as W1282X and that modulator function in rare variants can be quite distinct from classical findings based strictly upon F508del CFTR.

## Graphical abstract

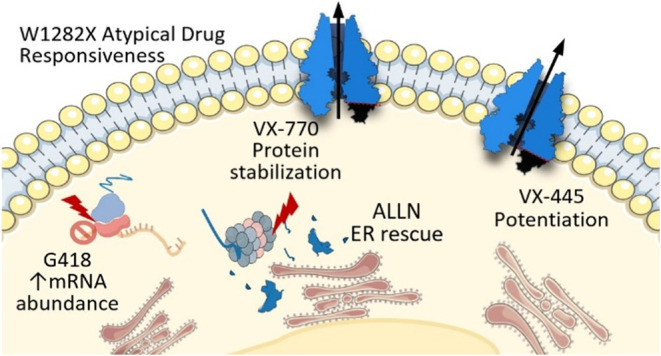

## Introduction

From the perspective of cystic fibrosis drug discovery, the W1282X CFTR presents a number of significant challenges. The variant represents a premature stop codon that foreshortens and destabilizes the peptide, blunts the half-life of the transcript,^1–3^ and omits the carboxy-terminal PDZ anchoring domain,^4–7^ all of which diminish the abundance of functional CFTR at the apical cell surface. These features pose significant barriers to high-throughput compound library screening and indicate the need for novel (non-CFTR) molecular targets involving pathways that improve mRNA expression, translation, or W1282X CFTR protein stability. In this report, we describe distinctive features of W1282X CFTR biogenesis, ion channel function, and pharmacologic rescue. In particular, we demonstrate the paradoxical effects of CFTR potentiator and corrector molecules when tested against the W1282X protein. To investigate the mechanism, we evaluated another variant (G551D CFTR) for which potentiation by VX-445 has been reported previously^8^ and observed acute CFTR stimulation by corrector molecules VX-809, VX-661, and VX-121. Using assays that monitor graded cAMP/PKA enhancement of function together with molecular dynamic simulations, we provide evidence for a mechanism involving changes in protein conformation and enhanced mutant CFTR ion transport following acute corrector therapy. We also show unconventional effects of both G418 and proteasome blockade on W1282X CFTR. Our studies emphasize the complexity of rare CFTR variants such as W1282X, add to previous work regarding this mutation,^5,9–11^ and show that special care must be taken when assigning CFTR modulator effects based on expectations from earlier studies of the prevalent F508del abnormality. The results also indicate that high-throughput compound library screening and standard protocols for testing modulator response need to account for unanticipated drug effects on CFTR folding and gating in order to avoid false-negative predictions of clinical benefit.

## Materials and Methods

### FRT Cell Lines

Isogenic Fisher Rat Thyroid (FRT) cell lines were established to stably express WT-, W1282X-, G551D-, or F508del CFTR, as well as parental (without CFTR) lines. Studies were performed as described previously,^12^ including CFTRs with a horseradish peroxidase-tagged extracellular loop 4.^13^ Coon’s modified Ham’s F12 (Sigma-Aldrich, St. Louis, MO) supplemented with 5% fetal bovine serum (GIBCO, Waltham, Massachusetts) and 100 μg/mL hygromycin B (Invitrogen, Waltham, Massachusetts) was applied to maintain cells, which were grown in a humidified incubator containing 5% CO_2_ at 37 °C. The cells were cultured without hygromycin B as monolayers on transwell filters (6.5 mm, 0.4 μm pore polyester membrane, Corning, 3470),^12^ or 96-well plates for FLIPR experiments, and pretreated for 24–72 h prior to short-circuit measurements.

### Primary Airway Epithelial Cells

The Emory Experimental Models Support Core provided nasal human airway epithelial cells as nasal brushes from healthy donors (non-CF volunteers) or patients with cystic fibrosis. The human use protocol was approved by the Emory University Institutional Review Board (#IRB00042577). Conditional reprogramming with an irradiated 3T3 fibroblast feeder layer and ROCK inhibitor^14^ was employed to propagate cells. Monolayers generated on collagen-coated filters were maintained with 5% CO_2_ at an air–liquid interface (ALI) for 2–3 weeks, with ALI medium changed every 2 days.^14,15^ FLIPR experiments utilized airway epithelial cells cultured in 96-well plate format under submerged conditions that included the presence of the notch inhibitor DAPT, as described previously.^16^

### Western Blotting

A RIPA lysis buffer (Thermo Fisher Scientific, Massachusetts) was used to prepare FRT cell lysates. Proteins were resolved by SDS-PAGE and then transferred to nitrocellulose membranes (Bio-Rad, Hercules, CA). Membranes were treated with 5% BSA in TBS-T (tris-buffered saline; 150 mM NaCl, 20 mM Tris-HCl pH: 7.4, 0.1% Tween 20) followed by 1 h incubation with CFTR UNC570, UNC769, and/or UNC596 (1:2500, 1:2000, and 1:2500, respectively; CFTR Antibody Distribution Program, University of North Carolina, Chapel Hill), MM13–4 (CFTR N-terminal; Sigma-Aldrich, St. Louis, MO), or 24–1 (CFTR C-terminal; R&D Systems, Inc., Minneapolis, MN) antibodies in TBS-T with 5% bovine serum albumin (BSA) solution. Loading control was β-actin. Membranes underwent three washings with TBS-T and were incubated for 1 h with polyclonal goat antimouse immunoglobulin-HRP (1:5,000; Dako, Denmark) in TBS-T with 5% BSA. The SuperSignal West Femto substrate (Pierce, Rockford, lL) was employed to detect the HRP signal, and visualization was performed on Chemidoc equipment (Bio-Rad, Hercules, CA). Imagelab band analysis software (Bio-Rad) was used to identify and assess band density. CFTR values were normalized to β-actin.

### Short-Circuit Current Measurements

Voltage clamp conditions (Physiologic Instruments, San Diego, CA) were used to evaluate the short-circuit current (*Isc*). Cells were pretreated 24 to 48 h prior to study, using DMSO or modulator compounds procured from Selleck Chemicals (Houston, TX) at concentrations described in the text. Measurement of CFTR channel activity followed acute addition of amiloride (100 μM), forskolin (5 μM for FRT cells, 10 μM for primary epithelial cells), VX-770 (5 μM), VX-661 (3 μM for FRT cells, 5 μM for primary epithelial cells), VX-809 (3 μM), VX-121 (3 μM), curcumin (40 μM), or CFTR_inh_-172 (10 μM) (Selleck Chemicals). A chloride gradient was utilized to enhance sensitivity (basolateral bathing solution contained 120 mM NaCl, 25 mM NaHCO_3_, 3.33 mM KH_2_PO_4_, 0.83 mM K_2_HPO_4_, 1.2 mM CaCl_2_, 1.2 mM MgCl_2_, and 10 mM d-glucose (pH 7.4) (Regular Ringer Buffer), while apical solution included 140 mM Na-gluconate, 1.2 mM NaCl, 25 mM NaHCO_3_, 3.33 mM KH_2_PO_4_, 0.83 mM K_2_HPO_4_, 1.2 mM CaCl_2_, 1.2 mM MgCl_2_, and 10 mM d-glucose (pH 7.4) (low chloride)). For FRT cell system experiments, the apical surface of monolayers was used for the addition of drugs. Primary airway epithelial experiments were performed with both apical and basolateral compound additions.

### Gene Expression

qPCR was employed to determine the relative levels of CFTR in FRT cells pretreated with DMSO or G418 according to the manufacturer’s specifications (qPCR QuantStudio5, Applied Biosystems, Foster City, CA), using 18S rRNA as an internal control. Droplet digital PCR (ddPCR; LSR QX200 system, Bio-Rad) was performed according to the manufacturer’s protocol. Total RNA collected from FRT cells was reverse transcribed using iScript Reverse Transcription Supermix (Bio-Rad). A target human CFTR primer-probe set (FAM labeled; assay ID = dHsaCPE5056656) and a reference rat-transferrin primer-probe set (HEX labeled; assay ID = RnoCPE5170129) were obtained from Bio-Rad. Analysis of droplet data was performed with QuantaSoft Software, and Microsoft Excel software was used for calculations. In each sample, target CFTR expression was normalized to that of rat transferrin expression.

### Molecular Dynamics Simulation

The ATP-free, unphosphorylated human (wild-type) CFTR membrane protein structure^17^ with inward-facing configuration (WT-CFTR) was taken from the PDB (PDB code 5UAK). 3D Builder in the Maestro program was used to establish a G551D CFTR construct. Elexacaftor (VX-445) ligand position in the G551D CFTR-VX-445 construct was identified by the alignment between the CFTR structure from 5UAK.pdb and the F508del CFTR structure in complex with VX-445 (8EIQ.pdb).

All three constructs (WT-CFTR, G551D CFTR, and G551D CFTR-VX-445) were appraised using the Protein Preparation Workflow available from Maestro (default settings). CHARMM-GUI Membrane Builder was applied in order to evaluate the membrane layer during simulation and comprised dimonounsaturated POPC [1-palmitoyl-2-oleoyl-glycero-3-phosphocholine] with 350 lipids in each leaflet. Protein orientation in the membrane was specified using a PPM 2.0 Web Server.^18^ The system^19–21^ was neutralized prior to setting the salt concentration to 0.15 M using NaCl. Simulation temperature was maintained at 310 K.

The AMBER99SB-ILDN^22^ force field was utilized for protein simulations, the Lipid21 force field for the membrane, and the Amber TIP3P water model. GROMACS input files for energy minimization, equilibration, and production simulations were generated using CHARMM-GUI.^20,23^ A steepest descent algorithm was employed for energy minimization.^24^ A six-step equilibration was initiated for NVT equilibration, for which position restraints on heavy atoms were employed together with gradual heating. NPT equilibration was followed to allow for model stabilization and adjustment of density. Production was run under NPT conditions.^25,26^

Three separate MD simulations with different seed numbers were performed for each construct (1 μs each), resulting in a total of 3 μs for each construct simulated. MD trajectories were analyzed using root-mean-square deviation (RMSD), and GROMACS tools were employed to calculate specific distances as a function of simulation time. According to the RMSD analysis, all simulations were converged (data not shown). We measured distances between the center of masses of the signature motif of NBD1 (LSGGQ) and the Walker A (1244–1252, GRTGSGKST) and Walker B (1370–1377, DEPSAHLD) motifs of NBD2, as well as between the signature motif of NBD2 (LSHGH) and the Walker A (458–466, GSTGAGKTS) and Walker B (572–579, DSPFGTLD) motifs of NBD1.

### Horseradish Peroxidase (HRP) Enzymatic Assay

CFTR in the plasma membrane was evaluated with an HRP tag in the fourth extracellular loop using cells expressing CFTR cDNA.^27^ Cultures received 48-h treatment with DMSO (Sigma-Aldrich, St. Louis, MO) or compounds including VX-809, VX-661, VX-445, VX-121 (Selleck Chemicals, LLC, Houston, TX), or G418 (Sigma-Aldrich, St. Louis, MO) as indicated. Wells were washed three times with PBS, followed by addition of the substrate (SuperSignal ELISA Femto substrate; Pierce, Rockford, IL) immediately before measurement. The luminescence signal was detected using a Flexstation 3 plate reader (Molecular Devices, San Jose, CA). The results are designated as the HRP fold increase after normalization to the DMSO control.

### FLIPR-Based Monitoring of Transmembrane Potential

CFTR anion conductance was identified by membrane depolarization using a FLIPR-based membrane potential dye assay (Molecular Devices, CA). The test was designed for high-throughput compound library screening and modeled after Laselva et al.^10^ Parental cells or FRT cells expressing W1282X CFTR were seeded onto 96-well plates (20,000 cells per well). 24 h post seeding, the cells received an additional 24 h of treatment with DMSO or 3 μM VX-809 and 5 μM VX-770. One hour prior to measurement, each well was given equal volumes of loading buffer (fluorescent dye diluted in regular Ringer buffer containing 0.83 mM K_2_HPO_4_, 3.33 mM KH_2_PO_4_, 1.2 mM CaCl_2_, 1.2 mM MgCl_2_, 25 mM NaHCO_3_, 120 mM NaCl, 10 mM glucose) and the plate was incubated for an additional hour at 37 °C. Immediately after incubation, the dye was omitted and the cells were examined in a fluorescence plate reader (Flexstation 3, Molecular Devices, CA). FLIPR dye prepared in low chloride Ringer solution was given at specific time intervals together with either DMSO, forskolin, modulators, or CFTR inhibitors. An excitation/emission wavelength of 525/565 nm was applied to monitor the membrane potential. Primary airway epithelial cells (nasal) from a W1282X/W1282X patient with CF were plated (50,000 cells per well) on collagen-coated clear-bottom black 96-well plates in ALI medium supplemented with 10 μM DAPT (Notch signal inhibitor, Selleck Chemicals, TX). After 3 weeks of ALI culture, with a change of medium every 2 days, the cells were examined. The resulting summary data represent an average of 4 repeats normalized to baseline (t = 0) following injection of low chloride buffer (100% FLIPR signal). The background from empty wells has been subtracted. Slopes were calculated using SoftMax Pro 7 software (Molecular Devices).

### Quantification and Analysis

Software packages Aquire and Analysis II (Physiologic Instruments, San Diego, CA) were employed to determine delta *Isc* values from Ussing chamber traces following each acute addition. SoftMax Pro 7 Software (Molecular Devices, CA) was used for FLIPR assay data. Imagelab 6.1 software (Bio-Rad, CA) was applied to quantify Western blot protein bands by densitometric analysis normalized to actin. All statistical analyses were performed with GraphPad Prism version 10 (GraphPad Software, San Diego, CA). Except when otherwise stated, data are presented as the mean ± standard deviation (SD). Comparisons between groups were conducted using one-way ANOVA, Brown-Forsythe test, repeated measures ANOVA, or Welch’s ANOVA, followed by Tukey’s or Bonferroni’s post hoc test as recommended by GraphPad software. Figure S1 was compiled using an unpaired parametric *t*-test (two-tailed). A *p*-value of <0.05 was considered statistically significant. Graphs were generated with the same software.

## Results

### Nonclassical Effects of CFTR Modulators on W1282X and G551D CFTR

The FRT cell system serves as a valuable resource for CF high-throughput drug screening^28–34^ and provides a means to test mechanisms that underlie disease pathogenesis, including studies directed toward CFTR modulator registration and label expansion.^35^ In FRT cells stably expressing W1282X CFTR, we observed that chronic administration of modulators leads to increased levels of the truncated CFTR protein (Figure 1A,B) and ion channel activity (Figure 1C–E). Interestingly, the W1282X CFTR protein expression was significantly augmented in cells chronically treated only with VX-770 (Figure 1B). This is in contrast to the situation for F508del, where VX-770 exerts minimal effect or may antagonize F508del CFTR correction in certain cell systems in vitro.^36–38^ Moreover, when drugs were added acutely, F508del corrector compounds functioned as modest activators of W1282X CFTR (Figure 1C,D,F). The agents therefore exert a “dual role” with regard to W1282X CFTR, and each can enhance either gating or maturational rescue. Robust residual function of truncated W1282X CFTR protein following curcumin treatment (shown in Figure 1E) has been reported previously as a feature that might be directed toward therapeutic intervention.^12,39^

**Figure 1:**
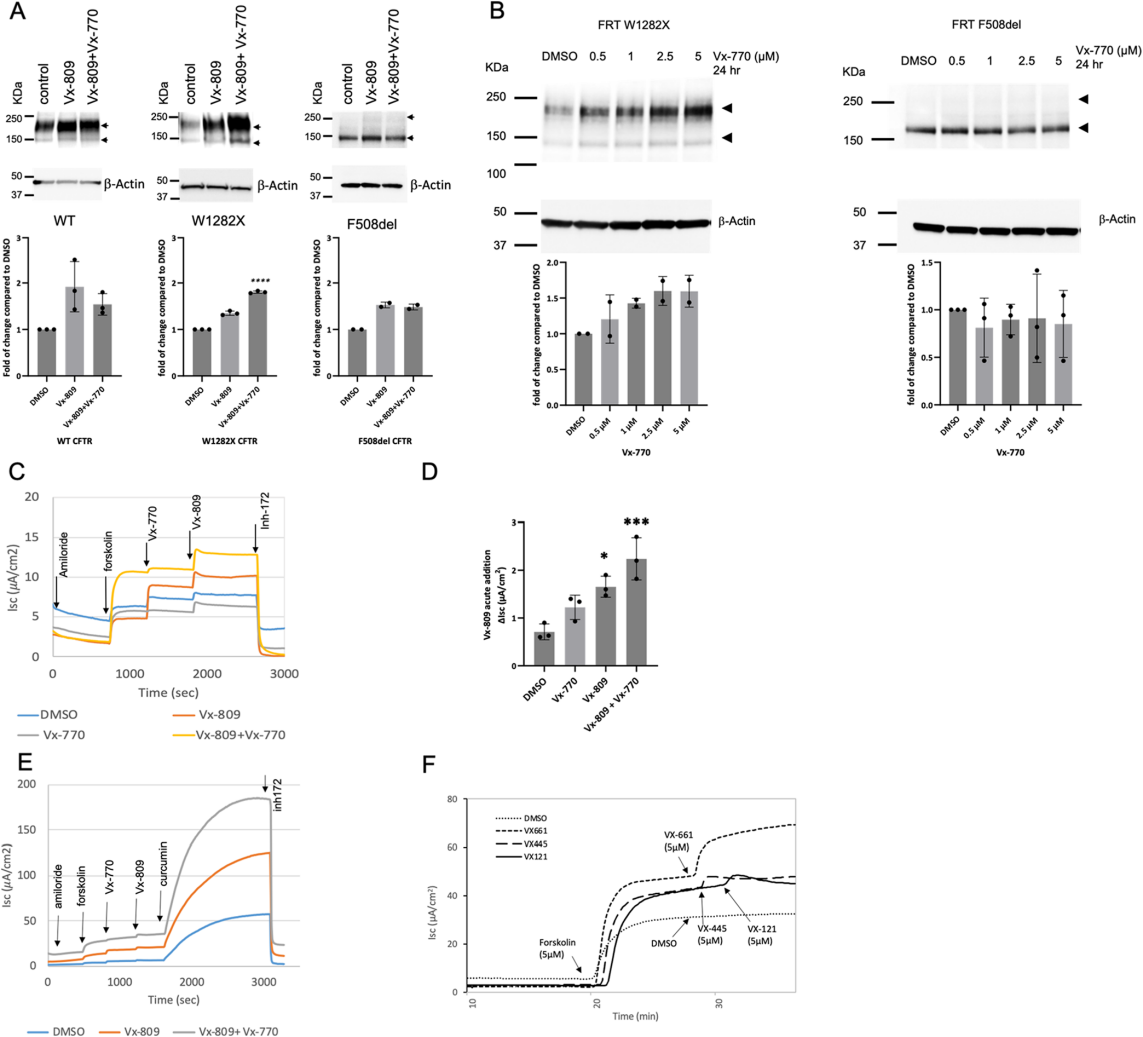
Chronic treatment with VX-770 augments W1282X CFTR steady-state expression, whereas acute corrector addition potentiates CFTR ion transport. (A) FRT cells expressing CFTR constructs were treated with 3 μM VX-809 and 5 μM VX-770 either alone or in combination for 48 h. Western blot (anti-CFTR mAb UNC596) demonstrates that expression of truncated CFTR is increased by combination treatment. Arrowheads indicate the position of the CFTR protein, including immature (Band B, lower) and mature (Band C, upper) glycoforms, respectively. Densitometry for W1282X CFTR reflects the total levels of Bands B and C combined. ****p < 0.0001 versus VX-809 alone. *n* = 3 biological replicates per condition. Statistics by the Bonferroni multiple comparisons method. No statistically significant difference was observed between VX-809 versus VX-809 plus VX-770 for F508del CFTR. (B) In the FRT cell model, chronic treatment (24 h) with VX-770 leads to an increased level of truncated W1282X but not F508del CFTR protein. *p* < 0.0001 for treatment with VX-770 across all concentrations versus DMSO vehicle. *n* = 2 biological replicates per condition. Statistics by Brown Forsyth Anova. When 24- or 48-h drug incubations were studied for effects on β-actin, results were comparable to vehicle control. When CFTR data were normalized to either β-actin or total protein loaded in each lane (i.e., 30 μg per condition), conclusions were unchanged. (C) Short-circuit current (*Isc*) was measured under voltage clamp conditions in FRT cells expressing W1282X CFTR and cultured as monolayers on transwell filters. Pretreatment was 48 h with DMSO, 3 μM VX-809, 5 μM VX-770, or 5 μM VX-770 plus 3 μM VX-809. W1282X CFTR channel activity was evaluated following acute addition of amiloride (epithelial sodium channel blocker, 100 μM), forskolin (CFTR activator via cAMP and protein kinase A, 5 μM), VX-770 (prototypic CFTR gating potentiator, 5 μM), VX-809 (CFTR maturational processing corrector, 3 μM), or CFTR inh172 (CFTR blocker, 10 μM). (D) Summary of short circuit from C. *p < 0.01 for acute activation by VX-809 after chronic VX-809 versus DMSO control. *n* = 3 biological replicates per condition. ***p < 0.0005 for acute activation by VX-809 after chronic VX-809 plus VX-770 versus DMSO control. *n* = 3. Statistics by Dunnett’s multiple comparison. (E) Curcumin strongly activates channel gating of W1282X CFTR,^12^ reaching levels approximately 50% of forskolin-activated WT-CFTR in the FRT model (not shown). Curcumin activation following chronic VX-809 was strongly enhanced in combination with chronic (48 h) VX-770. (F) VX-661, VX-121, and VX-445 are acute potentiators of W1282X CFTR. In Panel E, FRT cells expressing W1282X cDNA were pretreated with CFTR modulators VX-809 (3 μM) ± VX-770 (5 μM) for 72 h prior to short-circuit current measurement. In panels E and F, representative tracings are shown. Both experiments have been repeated with similar results. Small potentiator effects of VX-445 and VX-121 in Panel F were confirmed using 3–5 biological replicates per condition. Arrows depict the acute addition of indicated compounds. Vectoral chloride transport through CFTR (absent in parental cells) reflects the usefulness of this cell model as a means of monitoring CFTR activity at the cell surface. Results with inh172 (a CFTR blocker) also support the specificity of the findings for effects on the CF gene product. Error bars represent standard deviation.

In order to evaluate these findings in an additional, clinically relevant human cell model, we tested primary airway epithelia grown as polarizing monolayers at the air–liquid interface. W1282X leads to decreased CFTR mRNA, protein, and function compared to wild-type in this cell system.^40–42^ As reported previously, neither acute nor chronic modulator treatment resulted in a significant effect on W1282X CFTR in primary airway monolayers (Figure 2A).^9^ Because functional W1282X was barely detectable (<1 μA/cm^2^) under conditions examined here, we also investigated a CFTR variant (G551D) more strongly potentiated in primary airway cells. G551D CFTR exhibited pronounced activation when corrector molecules were given acutely in human nasal epithelia (Figure 2B), but not in cells expressing WT-CFTR (under saturating forskolin conditions; Figure 2C). Corrector compounds that have been FDA-approved for augmenting F508del CFTR maturational processing can elicit acute activation (i.e., resembling a potentiator) for both the W1282X and G551D variants (Figures 1C–F and 2B). The findings illustrate the complexity of modulator response among rare CFTR mutations and that important differences exist in comparison to effects on the F508del protein. In addition, G551D CFTR provides a useful and highly activatable model for evaluating mechanism(s) that may underlie acute CFTR potentiation by corrector molecules.

**Figure 2:**
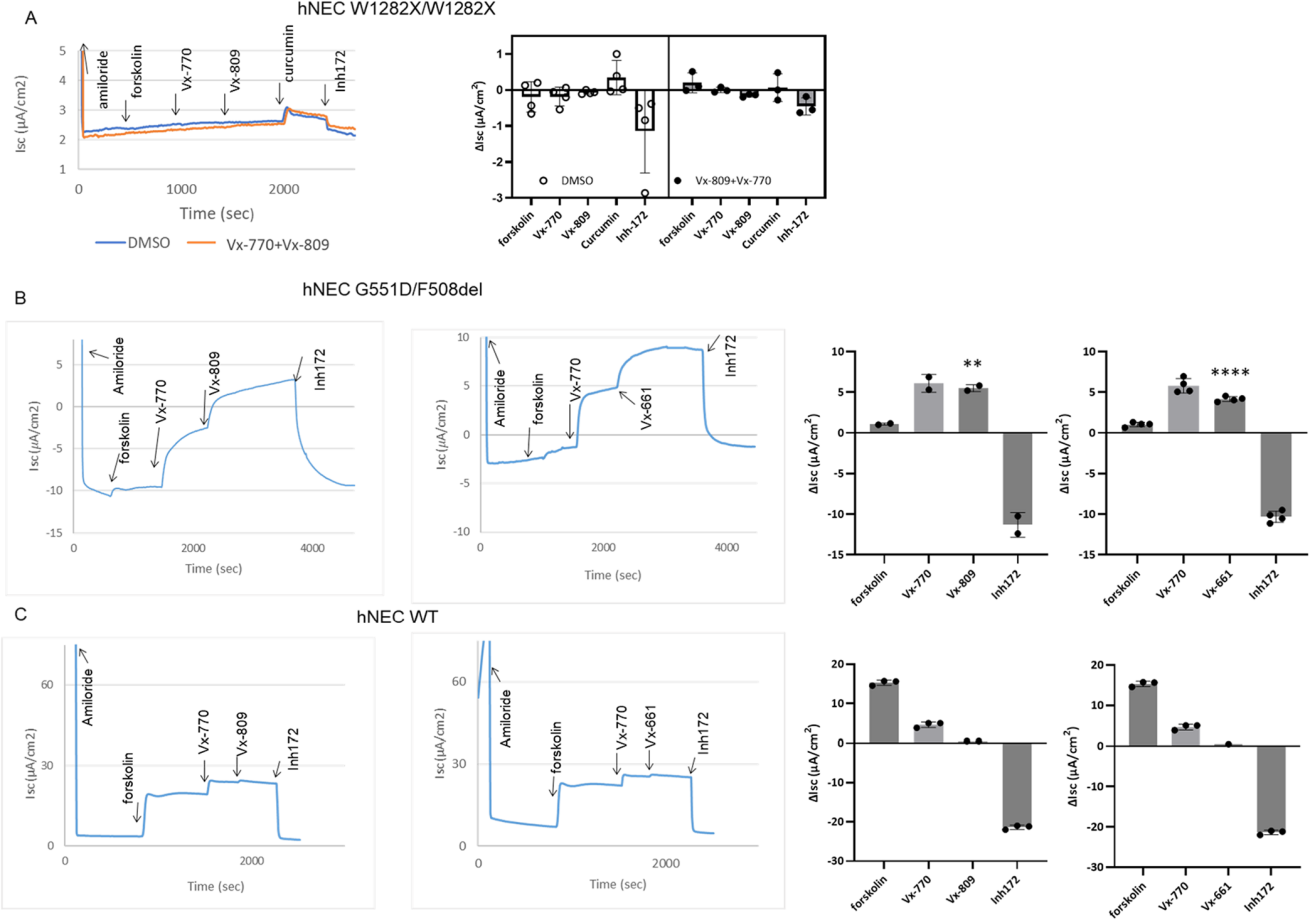
Studies of CFTR potentiation by VX-809 or VX-661 in primary human airway epithelial cells. Cells were cultured as monolayers on transwell filters, and short-circuit current (*Isc*) was measured under voltage clamp conditions to assess CFTR channel activity. Representative Ussing chamber traces and summary *Isc* data are shown. (A) Primary human nasal airway epithelial cells (hNEC) from a (W1282X/W1282X) CF patient were cultured at the air–liquid interface for 21 days and pretreated for 48 h with DMSO or 3 μM VX-809 together with 5 μM VX-770. No substantial forskolin or curcumin activation was observed. (B, C) Primary nasal airway epithelial cells from an individual encoding G551D/F508del (B) or a healthy (WT, wild-type) donor (C) were cultured as above for 21 days. ****p < 0.0001 for VX-661 versus previous compound addition. *n* = 4 biological replicates per condition **p = 0.0077 for acute VX-809 versus previous compound addition. *n* = 2 biological replicates per condition. VX-770 as a single agent differed from baseline (*p* < 0.02). Statistics by Tukey’s multiple comparison. Note: VX-809 and VX-661 (3 μM) are prototypic, clinically approved F508del CFTR maturational processing correctors. Because VX-661 and VX-809 do not acutely activate WT-CFTR (panel C), non-CF-related cation absorption or other (non-CFTR) anion channels are unlikely contributors to findings shown here or in Figure 1. Inclusion of saturating concentrations of amiloride prior to CFTR activation indicates that epithelial sodium channel (ENaC) dependent pathways do not contribute to the findings shown here. Results with inh172 (a CFTR blocker) further support the specificity of drug action on the CF gene product. Acute drug additions were otherwise as in Figure 1. hNEC: human nasal epithelial cells. Error bars represent standard deviation.

We hypothesized that treatment with corrector molecules would alter the folding of certain CFTR variants in a manner that facilitates gating. Because VX-445 and other corrector molecules strongly potentiate G551D CFTR, these compounds were utilized for further testing (Figure 3A,B). We found that acute administrations of CFTR corrector molecules shift the forskolin CFTR stimulation curve toward greater activation at lower levels of forskolin (Figure 3B). The result is compatible with an acute change in G551D CFTR conformation due to a corrector molecule and increased ion channel gating mediated by endogenous (including constitutive) levels of cellular cAMP and PKA (see also below).

**Figure 3:**
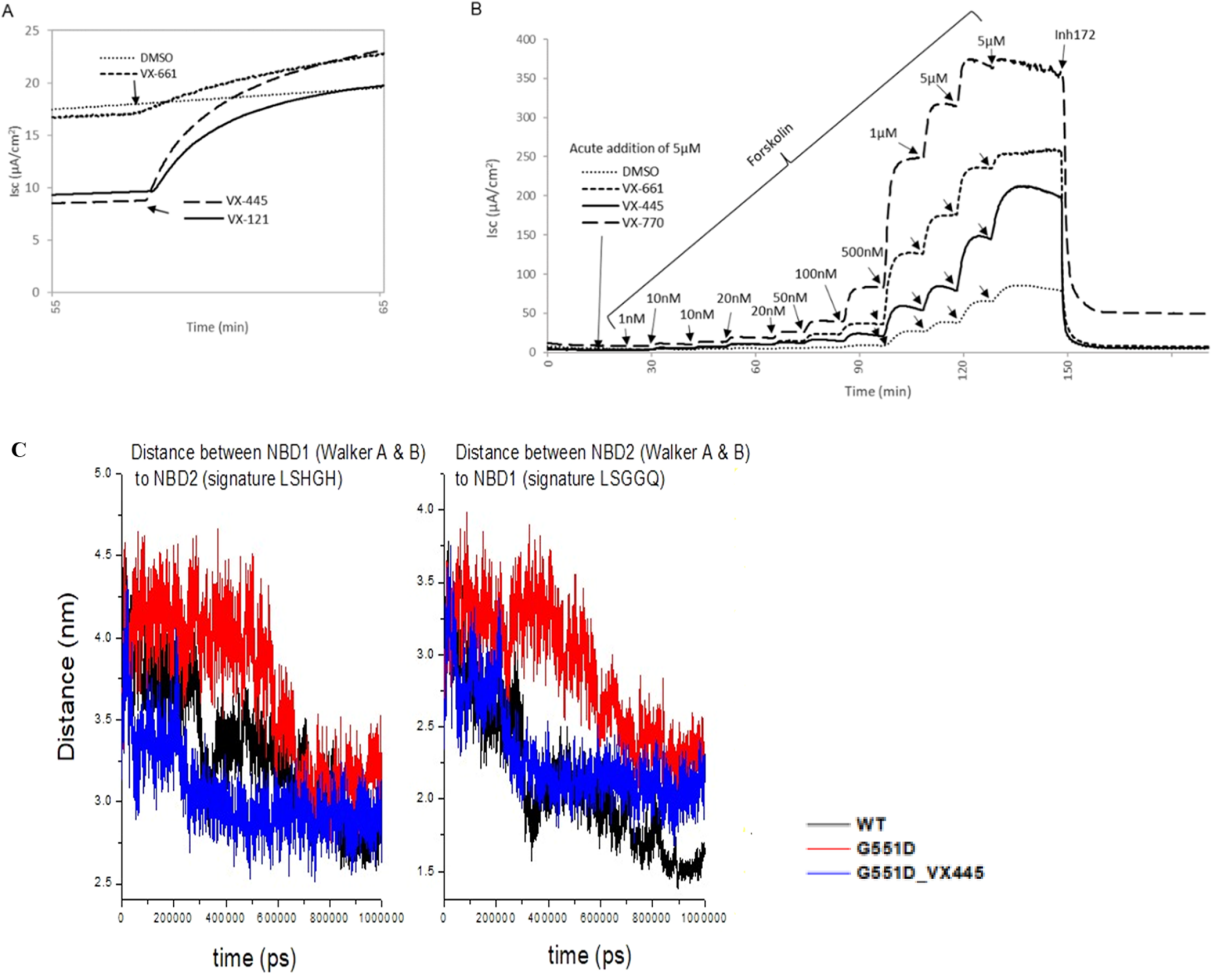
Acute additions of CFTR modulators enhance channel gating by cAMP/PKA. (A, B) VX-661 (5 μM), VX-445 (5 μM), and VX-121 (5 μM) acute treatment of G551D CFTR in FRT cells confers ion channel stimulation and shifts the forskolin activation curve toward greater activity, compatible with allosteric repositioning of the R-domain, NBDs, and lasso helices^43^ in a manner that promotes a greater likelihood of open configuration. The dose-dependent findings indicate effects on CFTR folding that elicit increased sensitivity to endogenous (including constitutive) cAMP/PKA. (C) Distances between the center of masses of the LSHGH motif of NBD2 and the Walker A and Walker B motifs of NBD1 and between the center of masses of the LSGGQ motif of NBD1 and the Walker A and Walker B motifs of NBD2 were averaged over three repeats of 1 ms. Molecular simulations of WT- and G551D CFTR in the presence or absence of VX-445 are shown.

To further develop this model, molecular dynamics (MD) simulations were performed. WT-CFTR, G551D CFTR, and G551D CFTR bound to VX-445 were investigated three times each for 1 s in the presence or absence of VX-445. Simulations were initiated from the cryo-EM human structure and “inward-facing” protein conformation (5UAK). Resulting trajectories were analyzed by measuring distances between the center of mass (COM) of the signature motif of NBD1 (LSGGQ) and the Walker A (1244–1252, GRTGSGKST) and Walker B (1370–1377, DEPSAHLD) motifs of NBD2 and between the center of masses of the signature motif of NBD2 (LSHGH) and the Walker A (458–466, GSTGAGKTS) and Walker B motifs (572–579, DSPFGTLD) of NBD1 as a function of simulation time. The signature Walker A and B motifs constitute binding sites for two ATP molecules upon NBD heterodimerization. Results presented in Figure 3C (distances averaged across the replicas) indicate that the space between each half of the ATP binding site in the WT and G551D+VX-445 constructs are smaller than in the untreated G551D protein, suggesting that VX-445 increases the tendency toward a configuration that resembles heterodimerization and in a manner that would favor channel opening.

### Studies of G418 as a Readthrough Agent to Rescue W1282X CFTR

To assess the aminoglycoside G418 for its effects on translational readthrough of CFTR encoding W1282X,^13,44,45^ we measured CFTR mRNA and probed full-length protein in FRT cells encoding the variant following 48-h drug treatment. G418 led to enhanced levels of W1282X mRNA and truncated protein (Figure 4A,B), and improved W1282X CFTR function (Figure 4D), at concentrations that did not promote detectable full-length CFTR (i.e., readthrough) by Western blot (Figure 4C) (see also ref 46).

**Figure 4:**
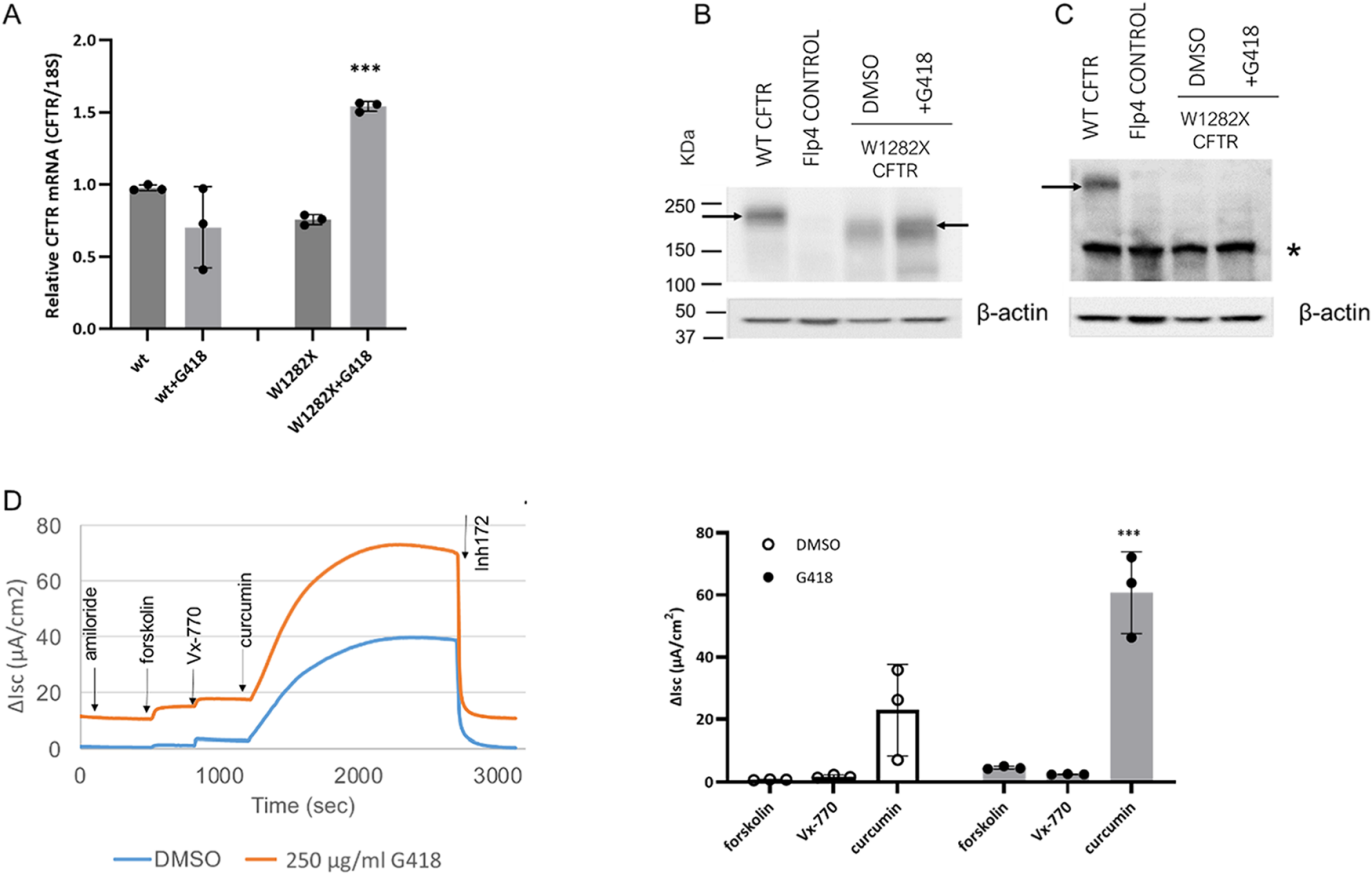
Truncated W1282X protein expression is enhanced by the readthrough agent G418 in FRT cells. (A) Incubation with 250 μg/mL G418 for 48 h in the FRT cell model significantly increased W1282X mRNA; ***p = 0.0003 for G418 versus DMSO in W1282X samples. *n* = 3 biological replicates per condition. Statistics by Bonferroni multiple comparisons. (B) Immunoblotting demonstrates increased CFTR W1282X truncated protein (right arrow) in cells treated with G418. Antibody (MM13–4) detects the N-terminus of CFTR. (C) CFTR W1282X was not detected by antibody 24–1 (directed against the C-terminus of CFTR); i.e., G418 treatment did not result in a measurable level of full-length W1282X protein (or readthrough) (arrow). *: nonspecific (background) band detected by antibody 24–1. Flp4 control in Panels B and C denotes an empty insertion site (no CFTR). (D) FRT W1282X CFTR cells were cultured as monolayers on transwell filters. 48 h prior to testing, cells were pretreated with DMSO or G418, as indicated. Representative Ussing chamber traces (left) and summary change in short-circuit current (*Isc)* (right) are shown in response to forskolin, VX-770, and curcumin; ***p = 0.0003 for curcumin following chronic G418 versus DMSO. *N* = 3 biological replicates per condition. Statistics were obtained by Bonferroni multiple comparisons. Acute additions of drugs to cell monolayers were otherwise as described in Figure 1. In (B and C), when data were normalized to either β-actin or total protein in each lane (i.e., 30 μg per condition), conclusions were unchanged. G418 together with CFTR modulators in primary airway epithelial cells has not shown substantial W1282X CFTR rescue.

### Proteasome Blockade Elevates Steady-State Levels of W1282X CFTR Protein

Misfolded F508del CFTR is confined to the endoplasmic reticulum (ER) and degraded by the ubiquitin-proteasome pathway. Inhibiting proteolysis of F508del CFTR using proteasome blockade leads to pre-Golgi entrapment of mutant protein but no significant CFTR maturation to the cell surface.^47,48^ In contrast, we found that treatment with a proteasome inhibitor (ALLN) increased both the W1282X CFTR protein (Figure 5, upper panel) and cell surface activity (lower panels). The result is distinct from other CFTR mutations, where proteasome inhibition does not enhance surface CFTR rescue.^48^

**Figure 5:**
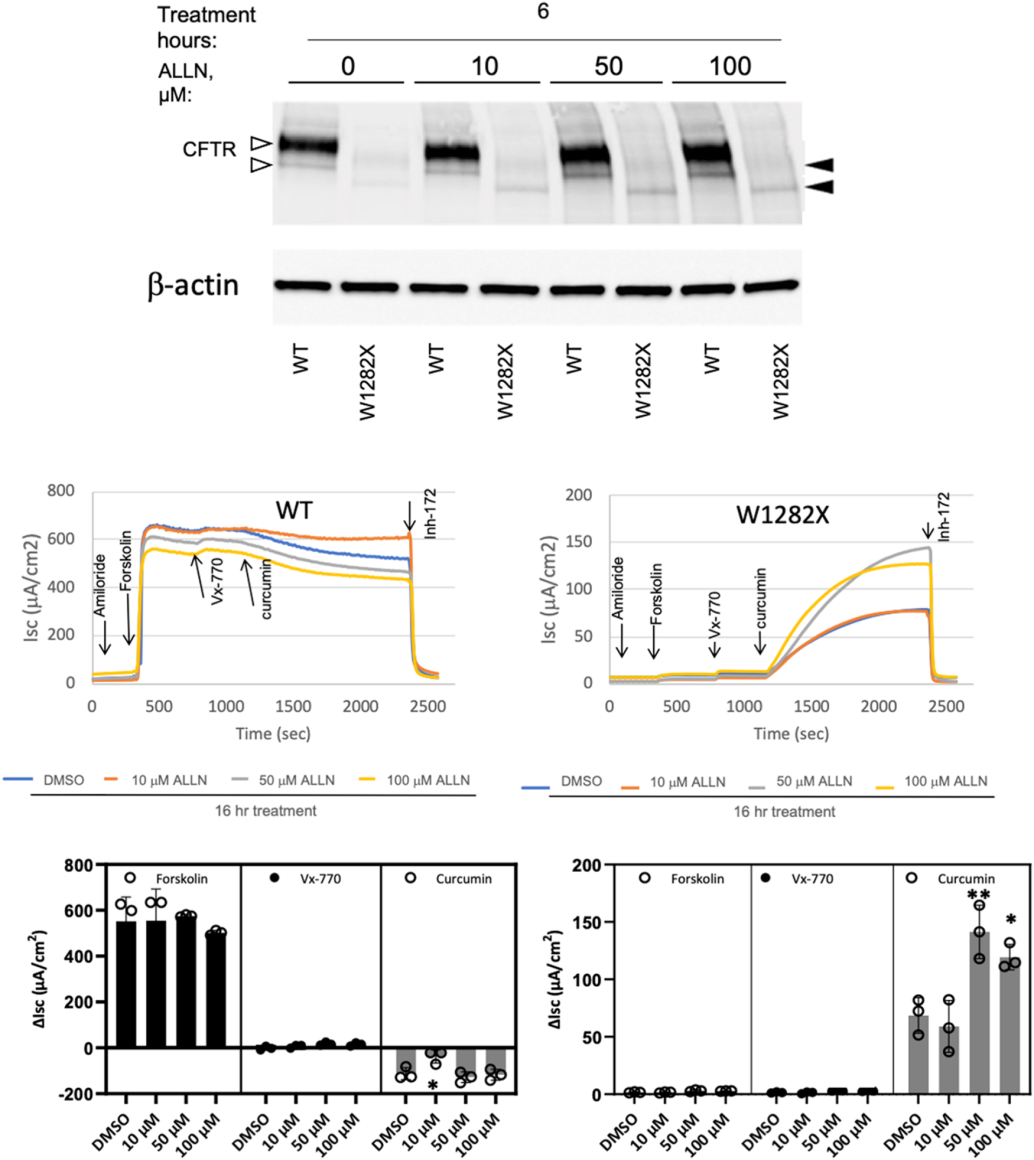
Proteasome inhibition confers increased W1282X CFTR protein synthesis and cell surface localization. FRT cells expressing WT or W1282X (truncated) CFTR were exposed to the proteasome inhibitor ALLN. Steady-state protein levels (upper panel) and functional assays (lower panels) are shown. Open arrowheads indicate WT-CFTR Band C (mature, fully glycosylated, upper arrowhead) or Band B (immature, core glycosylated, lower arrowhead). Closed arrowheads represent the corresponding W1282X (truncated) glycoforms. *p = 0.027 compared to DMSO control, *n* = 3 biological replicates per condition. **p = 0.0038 compared to DMSO control. *n* = 3 biological replicates per condition. Statistics were obtained by Dunnett’s multiple comparisons. Note that sensitive *Isc* measurements can be used to monitor surface-localized CFTR despite barely detectable protein by Western blot. Inhibition using inh172 indicates the specificity of these findings for W1282X CFTR-dependent activity (as opposed to other ion transport pathways). All lanes in the upper panels were loaded with 30 μg of the total protein. In the top panel, when CFTR data were normalized to either β-actin or total protein loaded in each lane, conclusions were unchanged. Modestly longer drug exposure times (16 versus 6 h) allowed optimal effects on W1282X function to be detected.

### Processing of W1282X CFTR to the Cell Surface

Because W1282X CFTR, when expressed as a truncated protein, lacks the COOH-terminal PDZ binding motif that otherwise facilitates cotranslational plasma membrane insertion and apical stability,^49–51^ CFTR proteins tagged with horseradish peroxidase (HRP) in the fourth extracellular loop were used to evaluate modulator-dependent routing to the apical cell surface^52^ (Figure 6). Plasma membrane localization of the truncated protein was increased following CFTR corrector, G418, or combination treatments (Figure 6A,B). Drug-induced plasma membrane localization was also enhanced for wild-type CFTR. In other studies shown above (Figures 1–5), vectoral chloride transport through CFTR was inhibited by the addition of inh172 specifically at the apical surface, indicating the activity of CFTR in the mucosal membrane. Specificity for CFTR is further supported by the complete absence of anion current in parental FRT cells (data not shown).

**Figure 6:**
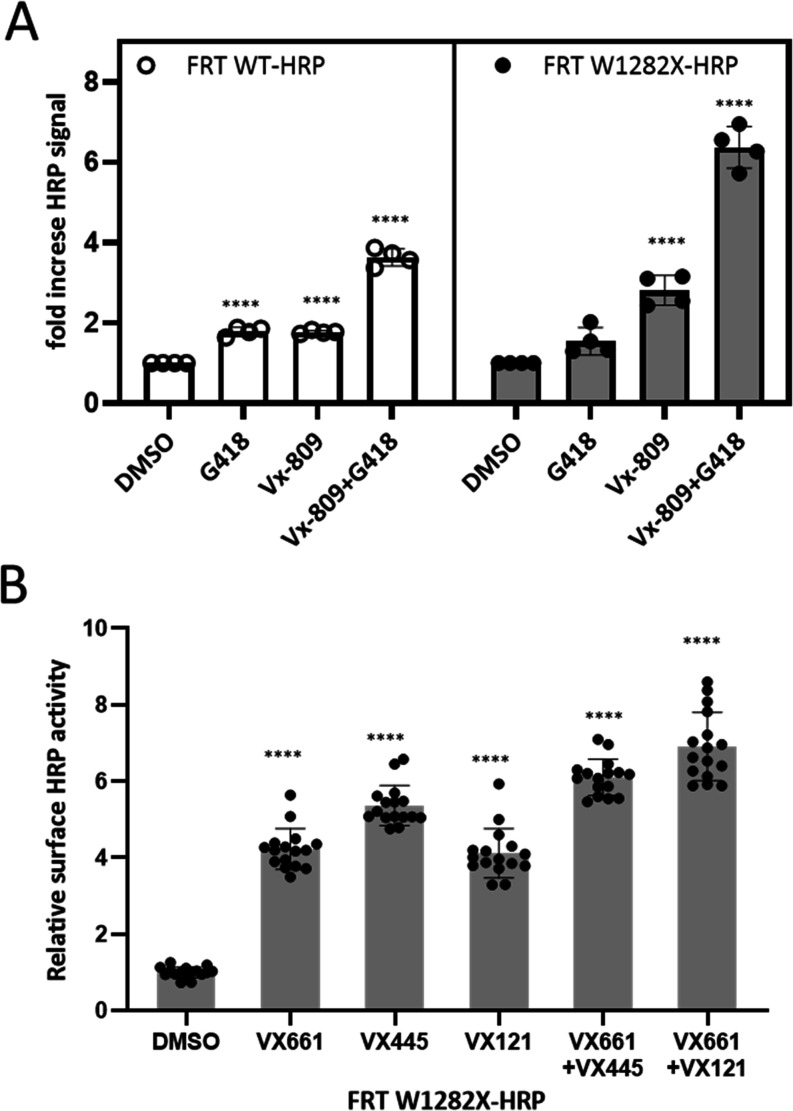
Cell surface expression of W1282X CFTR following chronic exposure to corrector molecules, G418, or a combination treatment. (A) HRP at the plasma membrane in FRT monolayers was quantified by exposure to a chemiluminescent substrate (Methods). Chronic (48 h) additions of clinically approved CFTR corrector molecules demonstrate strong enhancement of surface-localized CFTR, as judged by the HRP assay. FRT cells expressing either WT-CFTR-HRP or W1282X CFTR-HRP were treated with DMSO, 250 μg/mL G418, and/or 3 μM corrector molecules for 48 h. Significant increases in cell surface CFTR protein were observed in both FRT WT and W1282X cell lines compared to the DMSO control. ****p < 0.0001 for drug(s) compared with DMSO control. *n* = 4 biological replicates per condition. Statistics were obtained by Dunnett’s multiple comparison. FRT WT cell lines exhibit approximately 100-fold higher nonratioed HRP signal compared with W1282X. (B) Augmented W1282X CFTR surface expression following treatment with VX-661, VX-445, VX-121, or combinations. ****p < 0.0001 compared with DMSO control. *n* = 16 biological replicates per condition. Statistics were obtained by Dunnett’s multiple comparison.

## Discussion

In this report, we show that the CFTR potentiator ivacaftor (VX-770) and correctors lumacaftor (VX-809), elexacaftor (VX-445), tezacaftor (VX-661), and vanzacaftor (VX-121) augment W1282X CFTR steady-state expression and function. Interestingly, prototypic corrector agents also confer an acute ion transport activating effect (Figures 1–3). These results have significance, since they highlight the complexity of “theratyping” rare CFTR mutants (i.e., determining whether a specific potentiator or corrector agent is beneficial) and indicate the need to consider dual activity during drug screening programs that evaluate large numbers of rare variants.^53–56^ The finding that classically “pure” F508del corrector molecules can exhibit substantial acute potentiation of W1282X and G551D CFTR and that a “pure” potentiator can enhance steady-state protein levels of W1282X CFTR greatly broadens earlier findings of this type.^8,9,57,58^ Notably, VX-770 has been reported to blunt (not augment) correction of F508del CFTR,^38,59^ demonstrating that pharmacologic behavior using a rare variant such as W1282X can be quite different from findings observed with more common CFTR abnormalities. In the same context, our results show ways in which modulation of many rare CFTR variants might be erroneously interpreted if based strictly on effects shown previously for F508del. Data provided here further indicate (as judged by forskolin dose response and molecular dynamics studies) that acute corrector treatment confers CFTR conformational changes that can augment PKA sensitivity and CFTR activation.

We also evaluated a standard premature truncation codon (PTC) readthrough compound (G418) under conditions that failed to produce full-length W1282X CFTR in FRT cells. Instead, the drug unexpectedly and robustly increased the levels of both W1282X CFTR mRNA and functional truncated protein. Interventions shown previously to disrupt ribosomal activity may have a dramatic effect on mutant CFTR stability and maturation, and we speculate that a G418-dependent change in translational velocity,^60–62^ mRNA degradation, ribosomal collisions,^63^ and/or overall mRNA utilization^64–67^ may contribute to findings described here. While other CFTR premature stop codons are reliably rescued by G418, W1282X appears to require much higher dosing or more prolonged treatment to achieve stop codon suppression (unpublished observations). We show that a predominant effect of G418 may reflect increased W1282X mRNA abundance rather than the generation of full-length CFTR. The ion channel activity of the truncated protein (Figures 1–5 and S1), together with methods to augment W1282X mRNA, may have therapeutic implications for this particular PTC variant.^10,12,45^

Another unanticipated observation involved cellular degradative pathways associated with the W1282X CFTR. F508del and many other disease-causing cystic fibrosis variants are routed to endoplasmic reticulum-associated degradation (ERAD). Although F508del CFTR does not process in functional form to the cell surface following blockade of the proteasome,^47,68^ W1282X appears to behave differently, with proteasomal inhibition in FRT cells leading to enhanced W1282X CFTR activity at the plasma membrane. Based on results shown here, compound library screens to identify drugs that specifically rescue W1282X CFTR are anticipated to reveal new agents that enhance truncated protein levels with a negligible effect on readthrough, such as small molecules that block the proteasome.

As noted above, our present studies provide new information regarding barriers to overcoming the W1282X variant, including specific abnormalities not encountered for CFTR missense defects (Figure 7). Such obstacles include increased mRNA degradation, protein instability associated with an absent PDZ-binding domain (normally present at the CFTR carboxy terminus), and impaired ion channel activity of truncated CFTR (which can be strongly overcome by drugs such as curcumin).^12^ Our experiments utilized the FRT model, which has served as a mainstay for CFTR drug discovery (including all clinically approved CFTR modulators) but fails to reproduce key aspects of W1282X-related molecular pathogenesis. One limitation of the FRT line involves high levels of W1282X CFTR surface function far above what can be achieved in primary airway epithelia, resulting in strong ion transport activity that is absent in primary airway epithelial monolayers (Figure 2). Drug screening platforms utilizing W1282X FRT cells must therefore account for the abundant W1282X protein. While lower levels of W1282X CFTR in the plasma membrane of primary airway epithelium are due in part to nonsense-mediated mRNA decay, other recently appreciated defects associated with PTCs in cell-based systems (aberrant translational velocity, ribosomal stalling, queuing/collisions, and cotranslational degradation) should also be considered as possible contributors, and potential molecular targets, to improve W1282X CFTR.^62,63^ Based on the results shown here, inadequate W1282X CFTR surface protein can clearly be overcome without readthrough and despite a very large carboxy-terminal deletion (Figures 1 and 4–6).

**Figure 7:**
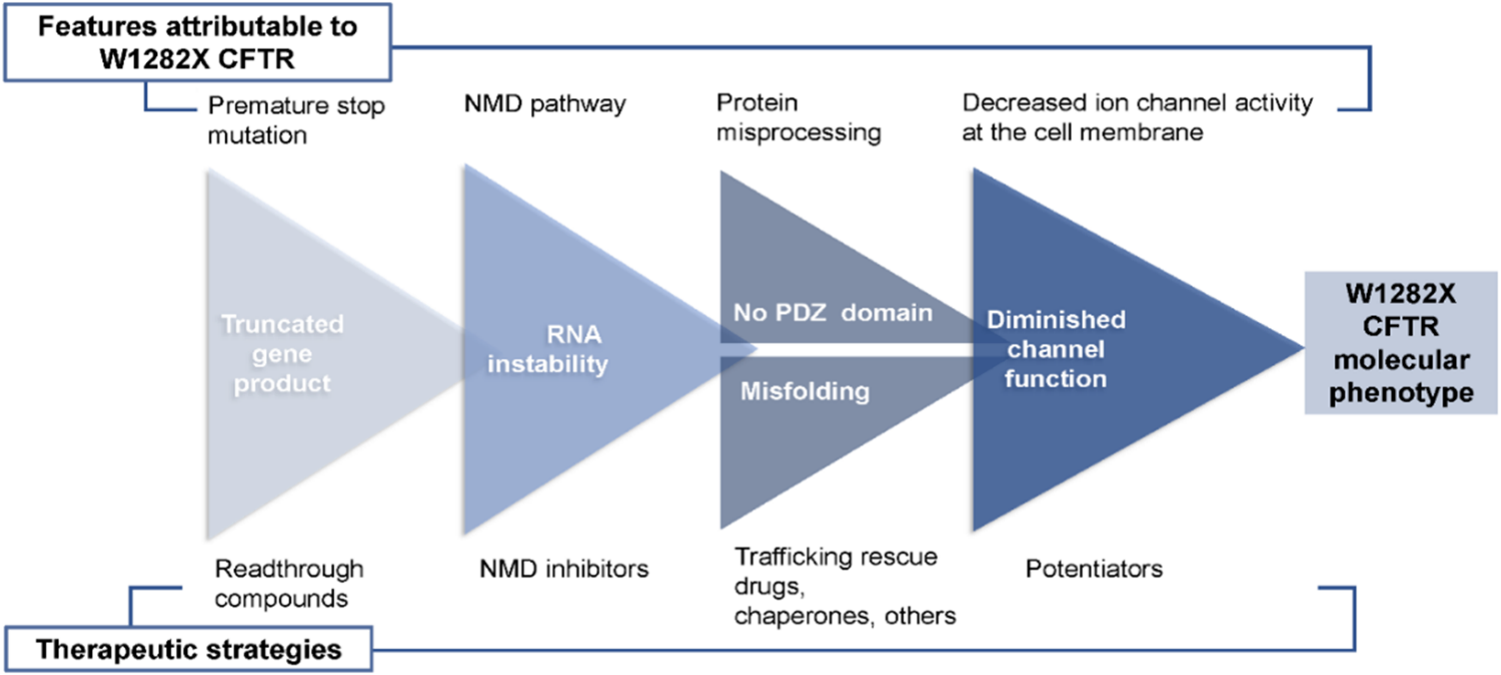
Challenges associated with W1282X CFTR drug discovery.

In a similar fashion, one can argue that drug screening protocols and/or confirmatory tests that employ primary airway epithelial cells should be applied early in the process of drug discovery to identify small molecules that specifically enhance truncated W1282X CFTR. For example, monitoring membrane polarity with FLIPR dye-based techniques has been shown to facilitate detection of CFTR in airway cells.^10^ A comparable approach might be used to test drug-responsive W1282X CFTR in primary human airway monolayers (Figure S1) and could be scalable to a 384-well (or larger) format for pharmaceutical drug screening. This strategy may furnish a means to address refractory W1282X CFTR defects, such as those depicted in Figure 7.

## Conclusions

In summary, findings presented here indicate the following: (1) activity profiles (e.g., potentiation versus correction) of CFTR modulator compounds based on F508del can be fundamentally different from those determined for rare mutations such as W1282X, (2) prototypic readthrough agents (G418) strongly enhance W1282X CFTR despite undetectable levels of readthrough, and (3) pathways that have largely been excluded as meaningful for CFTR rescue based on F508del (e.g., proteasome inhibition) may nevertheless strongly promote W1282X CFTR activity. The current studies also describe substantial complexity for PTC variants such as W1282X (summarized in Table S1), and they indicate the importance of incorporating key aspects of protein biogenesis and predicted folding configuration as part of next-generation drug screening programs. We believe that many CFTR variants (among over 2,000 reported to date) are likely to exhibit distinctive and/or idiosyncratic features such as those shown here. As a result, compounds viewed as unsuitable for treating certain CF genotypes and/or drugs designed for a specific purpose (potentiators, correctors, readthrough agents) can require independent re-evaluation to characterize effects on less well-studied CFTR abnormalities.^10,69^ In addition, large-scale drug analysis/theratyping programs that limit their protocols to chronic corrector treatment and acute addition of potentiators^70^ may fail to properly evaluate favorable drug responses and underestimate the potential for clinical benefit among rare forms of the disease. Our findings point to the need for more comprehensive profiling of rare CF variants than has been conducted previously, including the use of novel screening approaches to facilitate drug development for W1282X-related cystic fibrosis.

## Supplementary Material



## Notes

SI cover page; supplemental materials and methods; FLIPR-based W1282X CFTR detection in FRT and primary airway epithelial cells (Supplemental Figure S1); and summary of modulator responses (Supplemental Table S1) (PDF)
