# Supplementary material for: Barriers to the Pharmacologic Rescue of W1282X CFTR
Source: Biochemistry. 2025 Dec 12;65(1):123–35. doi: 10.1021/acs.biochem.5c00590 (PMC12781105; doi:10.1021/acs.biochem.5c00590)
Supplement: Supplementary file 1 [file bi5c00590_si_001.pdf]

Supporting Information

Title: Barriers to pharmacologic rescue of W1282X CFTR

Authors: Candela Manfredi[1], Andras Rab[1], Disha Joshi[1], Ashlyn G. Winters[1], JaNise J. Jackson[1], Sam Molina[2], Michael Koval[2], Netaly Khazanov[3], Madison Jacobson[1], Kathryn Oliver[1], Hanoch Senderowitz[3], Eric J. Sorscher[1*], Jeong S. Hong[1*]

[1] Emory University School of Medicine, Department of Pediatrics, Atlanta, Georgia, 30322, USA.

[2] Emory University School of Medicine, Experimental Models Core, Division of Pulmonary, Allergy, Critical Care and Sleep Medicine, Atlanta, Georgia, 30322, USA.

[3]Bar-Ilan University, Department of Chemistry, Ramat-Gan, 5290002, Israel.

*Email: esorscher@emory.edu

*Email: jeong.s.hong@emory.edu

- Supplemental materials and methods

- Supplemental Figure S1. FLIPR based W1282X CFTR detection in FRT and primary airway epithelial cells.

- Supplemental Table S1. Summary of modulator responses.

Supplemental Materials and Methods

Cell-based systems to screen for agents that rescue W1282X CFTR. Due to absence of intronic DNA and non-coding regulatory elements, as well as other limitations of W1282X FRT cell models, we tested miniaturized assays for monitoring W1282X CFTR in primary airway epithelia using a membrane potential-sensitive dye (FLIPR, Molecular Devices). FRT cells carrying W1282X CFTR (Figure S1A) or parental cells (without CFTR) (Figure S1B), and primary airway epithelial cells (Figure S1C) were evaluated. This general approach has shown favorable results in studies by others[1]. Cell monolayers incubated with FLIPR (after dilution in buffer to promote membrane hyperpolarization, see Methods) were transferred to low chloride medium and membrane potential measured in response to anion efflux through CFTR. Combination treatment with VX-809 and VX-770 in FRT W1282X cells showed increased FLIPR signal compared to parental FRT cells, indicating chloride transport through truncated CFTR[1]. Based on these findings, nasal airway epithelia from an individual with CF harboring W1282X/W1282X genotype was tested as part of a primary cell protocol that accounts for non-coding DNA, mechanisms that underlie nonsense-mediated decay, endogenous CFTR regulatory elements, etc. Primary airway cell monolayers were propagated in 96 well format, and membrane depolarization used to detect W1282X CFTR specifically following chronic treatment with VX-809/VX-770 (Figure S1C). Note that baseline polarity signal and behavior of the molecular probe (FLIPR) may be different in distinct cell types. For example, extent of FLIPR partitioning into the plasma membrane and the local (intra-membrane) environment can alter fluorescent signal, making quantitative comparisons between various cell models complex. These results emphasize importance of internal controls (e.g., +/- modulator treatment), as shown here.

1. Laselva O, Eckford PD, Bartlett C, et al. Functional rescue of c.3846G>A (W1282X) in patient-derived nasal cultures achieved by inhibition of nonsense mediated decay and protein modulators with complementary mechanisms of action. J Cyst Fibros 2020;19(5):717-727. (In eng). DOI: 10.1016/j.jcf.2019.12.001.

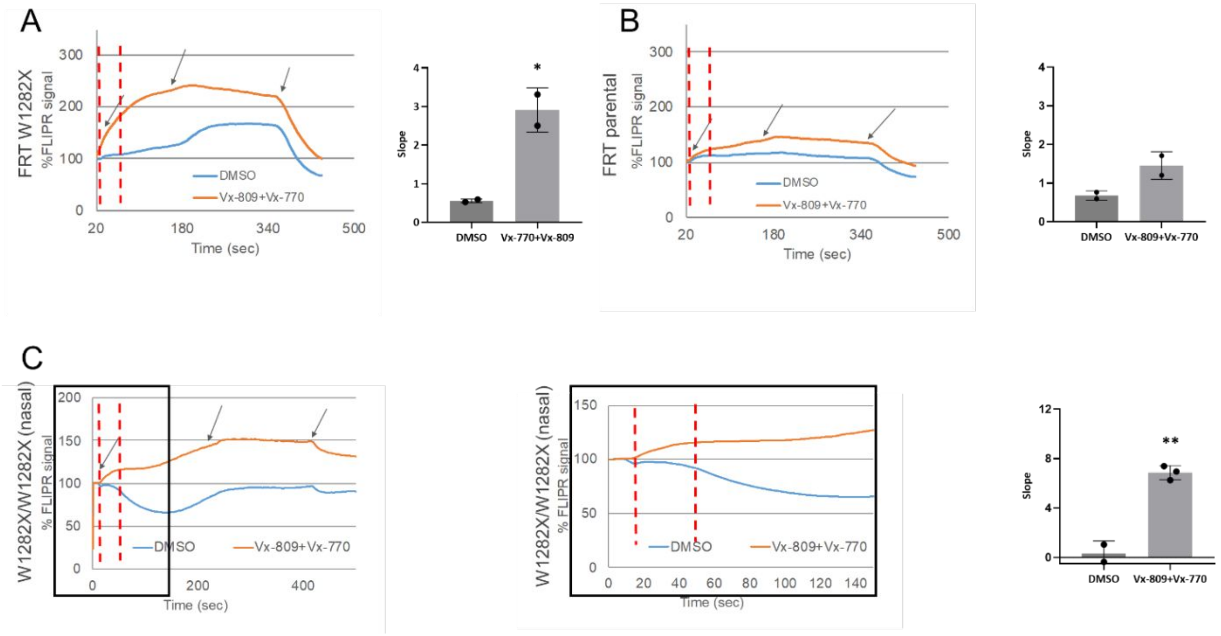

Figure S1. FLIPR based W1282X CFTR detection in FRT and primary airway epithelial cells.
CFTR analysis was performed in 96 well format using FRT cells expressing W1282X (**A**) or no CFTR (parental) (**B**) pretreated for 24 hours with DMSO (vehicle) or VX-809 (3 µM) + VX-770 (5 µM). FLIPR kinetic traces are shown on the left. Y axis represents relative fluorescence of FLIPR signal compared to no drug treatment. Left arrows: forskolin (5 µM); middle arrows: potentiator VX-770 (5 µM) +VX-809 (3 µM); right arrows: CFTR inhibitor 172 (10 µM). Slope of the interval defined between the red dashed lines (fluorescence tracing) are quantified in panels on right. (*p = 0.029, comparing DMSO vs 24 hr drug treatment, n=2 biologic replicates per condition.) Statistics by unpaired parametric two-tailed T test. Note that Inh172 appears to slightly (and non-specifically) inhibit FLIPR signal in absence of CFTR. (**C**) CFTR activity in 96 well format using primary nasal airway epithelia. W1282X/W1282X CF cells were pretreated for 24 hours with DMSO or VX-809 (3 µM) + VX-770 (5 µM). FLIPR kinetic trace is shown on left, and middle panel displays detailed view of initial time course (0-140 seconds). Right panel indicates summary data. Left arrows: forskolin (10 µM); middle arrows: potentiator VX-770 (5 µM) +VX-809 (3 µM); right arrow: CFTR inhibitor GlyH101 (25 µM) (**p =0.023, comparing DMSO vs 24 hr modulator treatment, n=3 biologic replicates per condition). Statistics were by unpaired parametric two-tailed T-test. Note that while low-level W1282X CFTR activation in primary airway cells may be difficult to monitor by short circuit current (Figure 2, and Haggie *et al*., 2017). other reports have successfully applied membrane potential sensitive techniques to document W1282X CFTR function (Laselva *et al.,*2020). Although these data indicate small molecules that overcome W1282X-related defects might be evaluated in rapid throughput fashion using primary cell systems, additional studies will be required to fully test that assertion.

Table S1. Summary of modulator responses. Potentiator function following acute addition of CFTR modulators was determined by short circuit current analysis (Materials and Methods). Corrector activity comprised incubating cells with modulators for increasing time intervals at 37°C, and monitoring both CFTR Band C and chloride transport. Examples of relevant citations are shown in parenthesis.

Modulators[1] used in current study		CFTR variants studied			
		Protein production Mutation Class 1[2]	Gating Mutation Class 3[2]	Protein processing Mutation Class 2[2]	Wild type CFTR UniProt P13569[4]
		W1282X CFTR rs77010898[3]	G551D CFTR rs202115599[3]	F508del CFTR rs113993960[3]	
Type I corrector	Lumacaftor (VX-809)	Corrector (Figure 6A)<br>Potentiator (Figures 1C, 1D)	Corrector**<br>Potentiator (Figure 2B)	Corrector[5,6]<br>No potentiator function[7]	Corrector (Fig 6B)*<br>Potentiator function not tested
	Tezacaftor (VX-661)	Corrector (Figure 6B)<br>Potentiator (Figure 1F)	Corrector**<br>Potentiator (Figures 2B, 3A)	Corrector[5,6]<br>Potentiator function not tested	Corrector*<br>Potentiator function not tested
Type III corrector	Elexacaftor (VX-445)	Corrector (Figure 6B)<br>Potentiator (Figure 1F)	Corrector*<br>Potentiator (Figures 3A, 3B)[8]	Corrector[6]<br>Potentiator[8,9,10]	Corrector*<br>Potentiator*[10]
	Vanzacaftor (VX-121)	Corrector (Figure 6B)<br>Potentiator (Figure 1F)	Corrector*<br>Potentiator (Figure 3A)	Corrector[8,11]<br>No potentiator function[12]	Corrector*<br>Potentiator function not tested
Potentiator	Ivacaftor (VX-770)	Corrector (Figure 1B)<br>Potentiator (Figures 1C, 1D)	Corrector function not tested<br>Potentiator (Figure 2B)[13,14]	No corrector function (Figures 1A, 1B)<br>Potentiator[7,13]	No corrector function<br>Potentiator (Figure 2C)[13,14]

*Unpublished data
**Findings include primary airway epithelial cells (not shown)

1. Baroni, D. (2025) Unraveling the mechanism of action, binding sites, and therapeutic advances of CFTR modulators: A Narrative review. *Curr Issues Mol Biol.* 47(2):119.
2. Types of CFTR mutations. https://www.cff.org/research-clinical-trials/types-cftr-mutations
3. SNPedia: https://www.snpedia.com/index.php/SNPedia
4. UniProt: https://www.uniprot.org/uniprotkb/P13569/entry
5. Fiedorczuk, K and Chen, J. (2022) Mechanism of CFTR correction by type I folding correctors. *Cell* 185:158-168
6. Fiedorczuk, K and Chen, J. (2022) Molecular structures reveal synergistic rescue of Delta508 CFTR by Trikafta modulators. *Science* 378: 284-290
7. Kopeikin, Z et al. (2014) Combined effects of VX-770 and VX-809 on several functional abnormalities of F508del-CFTR channels. *J Cyst Fibros*. 13(5):508-514
8. Veit, G., Vaccarin, C., and Lukacs, G. L. (2021) Elexacaftor co-potentiates the activity of F508del and gating mutants of CFTR, *J Cyst Fibros.* 20, 895-898
9. Shaughnessy CA, Zeitlin PL, Bratcher PE. (2021) Elexacaftor is a CFTR potentiator and acts synergistically with ivacaftor during acute and chronic treatment. *Sci Rep.* 11:19810
10. Laselva, O., Bartlett, C., Gunawardena, T. N. A., Ouyang, H., Eckford, P. D. W., Moraes, T. J., Bear, C. E., and Gonska, T. (2021) Rescue of multiple class II CFTR mutations by elexacaftor+tezacaftor+ivacaftor mediated in part by the dual activities of elexacaftor as both corrector and potentiator, *Eur Respir J.* 57 (6):2002774.
11. Langevald G and Bear C. (2025) EPS7.01Comparing elexacaftor and vanzacaftor: impact on CFTR functional rescue and protein maturation. *J Cyst Fibros.* 24 Suppl:S60
12. Kolski-Andreaco A et al. (2025) (R)-vanzacaftor potentiates BKCa channels in the absence of CFTR correction or potentiation. *Am J Physiol Cell Physiol*. 329(4):C1130-C1138
13. Van Goor, F., Yu, H., Burton, B., and Hoffman, B. J. (2014) Effect of ivacaftor on CFTR forms with missense mutations associated with defects in protein processing or function, *J Cyst Fibros.* 13, 29-36
14. Yu, H., Burton, B., Huang, C. J., Worley, J., Cao, D., Johnson, J. P., Jr., Urrutia, A., Joubran, J., Seepersaud, S., Sussky, K., Hoffman, B. J., and Van Goor, F. (2012) Ivacaftor potentiation of multiple CFTR channels with gating mutations, *J Cyst Fibros.* 11, 237-245
